# A network meta-analysis to evaluate the efficacy of traditional Chinese medicine on intestinal flora in patients with gastrointestinal cancer

**DOI:** 10.3389/fgene.2022.1069780

**Published:** 2022-11-28

**Authors:** Niran Feng, Shurui Wang, Chengjiang Liu, Zixin Xu, Zhijie Song, Kunyang Li, Zhifeng Yu

**Affiliations:** ^1^ Graduate College, Tianjin University of Traditional Chinese Medicine, Tianjin, China; ^2^ Department of Gastroenterology, Anhui Medical University, Hefei, China; ^3^ Graduate College, Shaanxi University of Traditional Chinese Medicine, Xi’an, China; ^4^ Department of Chinese Medicine, School of Chinese Medicine Engineering, Tianjin University of Traditional Chinese Medicine, Tianjin, China

**Keywords:** gastrointestinal cancers, intestinal flora, systematic review, treatment, traditional Chinese medicine (TCM)

## Abstract

**Background and Purpose:** Traditional Chinese medicine (TCM) can regulate intestinal flora so as to affect the occurrence, progression, and prognosis of gastrointestinal cancer. According to clinical studies, TCM oral administration, TCM external treatment, and TCM injections, can adjust intestinal flora disorders in patients with gastrointestinal cancer. This network meta-analysis aims to evaluate the effect of three treatments on the intestinal flora in gastrointestinal cancer patients.

**Methods:** This meta-analysis was registered in PROSPERO (CRD42022332553). Six electronic databases, namely CNKI, Wanfang, CSTJ, PubMed, Cochrane Library, and EMBASE, were searched from their inception to 1 April 2022. We identified randomized controlled trials (RCT) used to compare the efficacy of three TCM treatment methods—oral administration, external therapy and injections—on the intestinal flora in gastrointestinal cancer patients. The main outcome indicators were *Bifidobacteria*, *Lactobacilli*, *Escherichia coli*, and *Enterococci*. Stata (15.1) and the Cochrane risk of bias assessment tool were employed.

**Results:** We identified 20 eligible RCTs with a total of 1,774 patients. According to network meta-analysis results, TCM injection plus common treatment (CT) or oral administration of TCM plus CT was superior to CT alone for supporting *Bifidobacterium*. In supporting *Lactobacillus*, TCM injection plus CT demonstrated more obvious effect relative to oral administration of TCM plus CT; TCM injection plus CT was more effective than CT only; and oral administration of TCM plus CT was superior to CT only.The inhibitory effect of TCM injection plus CT on *Escherichia coli* was better compared with CT only. In terms of inhibiting *Enterococci,* oral administration of TCM plus CT was superior to CT only.The difference in efficacy among the above treatments was statistically significant. In the SUCRA probability ranking, TCM injection plus CT had the best ranking curve among the three treatments and was the most effective in supporting *Bifidobacteria* (Sucra = 90.08%), *Lactobacilli* (Sucra = 96.4%), and regulating *Escherichia coli* (Sucra = 86.1%) and *Enterococci* (Sucra = 87.1%).

**Conclusion:** TCM injections plus CT is the most effective therapy in balancing the intestinal flora of gastrointestinal cancer patients. However, the current results deserve further validation through high-quality research.

**Systematic Review Registration**: http://www.prisma-statement.org/, identifier 10.1136/bmj.n71.

## 1 Introduction

Gastrointestinal (GI) cancers, including gastric cancer, colon cancer, and colorectal cancer (Soleimanpour et al., 2020), are among the most common cancers (Wang et al., 2020a), accounting for approximately 26% of total cancer incidence and about 36.4% of cancer-related deaths (Arnold et al., 2020). Recently, the incidence and mortality of GI cancers have been increasing (Bray et al., 2018), so exploring the protective factors and risk factors for the occurrence and development of GI cancers will be conducive to effectively preventing and treating these cancers. Clinically, GI cancers are usually treated by radiotherapy, chemotherapy, surgery, drugs, and immunotherapy, while TCM, generally considered as an adjuvant therapy combined with radiotherapy and chemotherapy, plays an effective anti-tumor role by inducing tumor cell apoptosis and inhibiting tumor angiogenesis (Wang S. et al., 2021). At the same time, it decreases the gastrointestinal reactions caused by radiotherapy and chemotherapy (Zhang et al., 2021). However, with the in-depth study of the relationship between TCM and intestinal flora and gastrointestinal cancers, we found that TCM can adjust intestinal flora, promote beneficial bacteria to produce more Short-chain fatty acids (SCFAs) (Martin-Gallausiaux et al., 2021), mainly including acetate (C2), propionate (C3) and butyrate (C4), and improve the microenvironment of the gastrointestinal tumors, thereby having a certain beneficial impact on the occurrence, development, and prognosis of GI cancers (Sivan et al., 2015). In addition, intestinal flora also has a therapeutic effect on radiation enteritis caused by radiotherapy (Jian et al., 2021). Therefore, we believe that TCM can treat patients with gastrointestinal cancers by regulating intestinal flora in multiple ways.

Human intestinal microbes constitute a complex ecosystem, with around 800 species and more than 7,000 bacterial strains (Ley et al., 2006). In the intestine, symbiotic microorganisms are dynamic, which can maintain intestinal stability and inhibit pathogen colonization. When the balance is broken, the intestinal mucosal barrier and immune function will be undermined, leading to additional pathogenic factors, which are risk factors for colorectal cancer as well (Si et al., 2021).Clinical studies have found significant changes in the structure and characteristics of the intestinal flora in gastrointestinal cancer patients (Ferreira et al., 2018). Additionally, intestinal flora affects the absorption of anticancer drugs and correlates with the prognosis of these patients (Wertman et al., 2021). The pathological mechanisms by which intestinal flora affects colorectal cancer are currently thought to be achieved through multiple pathways, such as the induction of inflammation and immunity (Meng et al., 2018). Notably, intestinal pathogenic bacteria can drive tumorigenesis by shaping the tumor microenvironment or forming biofilms, such as *Bacteroides*, *Escherichia coli*, and *Clostridium difficile*, which can secrete a variety of virulence factors that damage intestinal epithelial cells and trigger chronic inflammatory responses, and develop into colorectal cancers (Hayase and Jenq, 2021). Meanwhile, some intestinal probiotics can directly produce tumor suppressive substances or enhance related antigens to achieve anti-tumor effects (Song et al., 2021). The ferritin produced by *Lactobacillus* casei ATCC334, for instance, can act as a tumor suppressor through the JNK signaling pathway (Konishi et al., 2016). Therefore, we consider how to balance the environment of intestinal microbiota deserves further exploration.

Among many pathogenic bacteria, *Escherichia coli* and *Enterococcus*, belonging to neutral bacteria, are not pathogenic when their population is within a certain range, however, an excessive number of these bacteria may produce *Enterotoxin* that are highly pathogenic (Wassenaar, 2018; Alhinai et al., 2019). *Bifidobacteria* and *Lactic acid bacteria*, as probiotics, produce a large amount of SCFAs, which are beneficial to intestinal health (Zaharuddin et al., 2019). Because of their large number, more in-depth basic research, and easy clinical detection, when intestinal pathology changes, the flora changes significantly, so they are commonly used as clinical indicators for evaluation of intestinal flora (Kuugbee et al., 2016).

In fact, the TCM adjuvant therapy for cancer has achieved a remarkable clinical efficacy (Wang S. et al., 2020c). Nowadays, the main TCM therapies commonly used in the clinics are oral therapy, external therapy and injection therapy (Huang et al., 2018). The classification is based on different routes of administration. Oral treatments of TCM are absorbed through the gastrointestinal tract, external treatments of TCM are absorbed through the skin and mucosa by physical therapy or enema, and TCM injections are the components directly enter the bloodstream. To the best of our knowledge, most of the previous studies have focused more on TCM oral administration, and less on external treatments and TCM injections. It has been shown that TCM can inhibit the development of cancer by regulating intestinal microbes (Chen et al., 2021). XiaoYao decoction (a medicinal diet with Ginseng, Atractylodes and Fushen as the main ingredients), for instance, can increase the abundance of *Bacteroides, Lactobacillus*, and *Proteobacteria*, and reduce the abundance of *Desulfovibrio* and *Rickerella* (Zhang Z. et al., 2020).

Given that the evidence in the current literature not able to determine which one is the most effective Therefore, we tried to select the best treatment by counting and analyzing the changes of 4 indicators in intestinal flora after the application of three TCM treatments in the previous literature. To date, no meta-analysis has been conducted to compare the effects of CT in combination with each of these three TCM methods on intestinal flora in gastrointestinal cancer patients. We present the paper on the basis of the checklist of the extended PRISMA for network meta-analysis.

## 2 Materials and methods

This meta-analysis was registered in PROSPERO (CRD42022332553).

### 2.1 Search strategies

We searched three English databases (PubMed, Cochrane Library and Embase) and three Chinese electronic databases (CNKI, Wanfang and Chinese Science and Technology Journal Database). The search period started from the establishment of the database until 1 April 2022.

Our search strategy contains comprehensive terms in the English database as follows: (Medical, Chinese traditional or Chinese medicine) and (gastric or colorectal or colorectal or gastrointestinal tumors) or (intestinal flora or gut microbes or *Bifidobacterium* or *Lactobacillus* or *Escherichia* coli or *Enterococcus*). A comprehensive search with subject terms, joint keywords and free words was conducted according to different databases to ensure the systematization and integrity of the search.

### 2.2 Inclusion standards

(1) The symptoms and clinical indicators of patients were in accordance with the newly compiled guideline *The Diagnostic Criteria of Gastrointestinal Tumors.*
(2) Randomized controlled trial.
(3) The control group was treated with CT, and the treatment group with one of three TCM intervention methods, namely CT + Oral administration of TCM, CT + external therapy of TCM (e.g., enema of TCM, acupoint catgut embedding, cutaneous scraping therapy, acupuncture, and moxibustion), and CT + TCM injection.
(4) The observation indicators are the numbers of *Bifidobacterium*, *Lactobacillus*, *Escherichia coli* and *Enterococcus.* At least one result was available in the literature.
(5) The intestinal microbiota numbers in fecal samples of patients can only be analyzed by 16SrDNA sequencing.

### 2.3 Exclusion standards

(1) Literature review, animal experiment, experience summary and other types of literature are excluded.
(2) Patients with non-simple gastrointestinal cancer.
(3) The treatment group did not meet the requirements of combined TCM and common treatment or did not use one of the 3 treatment methods of TCM, or the control group was treated with TCM.
(4) Articles with multiple publications and those with full text unavailable or with incomplete data were excluded.
(5) None of the four selected indicators of intestinal flora (*Bifidobacteria*, *Lactobacilli*, *Escherichia coli*, and *Enterococci*) was found in the outcome indicators of RCT.

### 2.4 Types of outcome measures

The outcome indicators of this study were determined based on the frequency of outcome indicators in the articles involved and the 2020 AGA clinical practice guidelines. The main outcome indicators are as follows: 1) *Bifidobacteria* and *Lactobacilli* (increased number) and 2) *Escherichia coli* and *Enterococci* (decreased number).

### 2.5 Literature screening and data extraction

According to the search strategy, relevant literature was found in the database and the bibliography was exported. Duplicate literature was excluded using Endnotex9 software.The literature that met the inclusion criteria were downloaded for comparison, and the full text was ultimately read for exclusion.

Two reviewers (Niran Feng and Kunyang Li) independently searched the database and the selected articles. If there was any disagreement between them, a third party (Shurui Wang) would participate in the discussion and propose a solution to resolve their differences. Furthermore, the references in the selected studies were examined to incorporate literature missing from the main studies.

Data extraction criteria included: first author, publication year, country, title, number of cases, treatment duration, intervention measures in both the experimental group and the control group, and treatment results.

### 2.6 Quality assessment

Two researchers (Niran Feng and Zixin Xu) independently assessed the literature according to the inclusion and exclusion criteria. A third party participated in the discussion and decided whether there was any objection. We used the Revman software 5.2 and the “bias risk assessment” tool recommended by the Cochrane manual as the evaluation index for the quality assessment for all included studies. We evaluated the content of the literature with high risk, low risk, and unknown risk. In the case of incomplete data during the evaluation process, we obtained data by contacting the authors.

### 2.7 Statistical investigation

Considering the data of the four intestinal microbiota as continuous variables, the weighted mean difference (WMD) and 95% CI were used as effect size indicators for continuous variables. The difference was considered statistically significant, when the confidence interval (CI) was set to 95% and 0 was excluded. The data extracted from the article were ranked for efficacy and ranked cumulative probabilities using stata15.0 (Stata Corporation, College Station, TX, United States). Heterogeneity was assessed using funnel plots, where I^2^ values greater than 50% represented considerable statistical heterogeneity. In addition, data processing, network link graph, forest graph and surface under the curve ranking (Sucra) were completed sequentially.

## 3 Results

### 3.1 Literature search of the included studies

**FIGURE 1:**
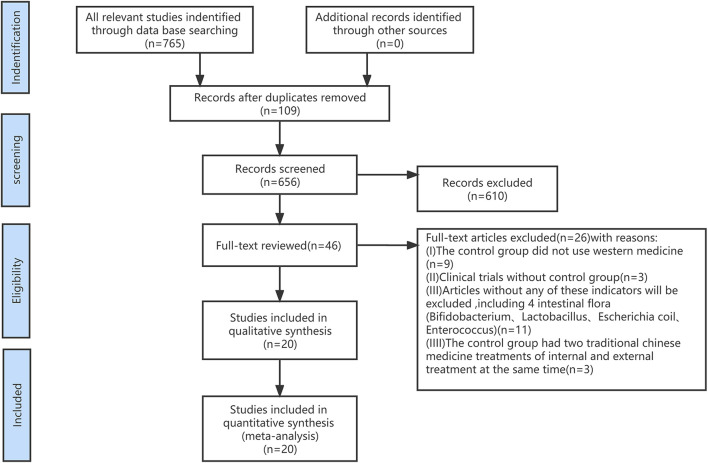
The process of literature filtering.

First, we screened out 765 articles and eliminated 109 duplicates according to the search criteria. Next, after reading the titles and abstracts, another 610 references were excluded. Finally, the remaining 46 articles were read and 20 eligible RCTs were included (Figure 1). The 20 RCTs comprised a total of 1,774 patients, including 888 in the treatment group and 886 in the control group. All included studies were conducted in China, with a sample size range of 30–109 entries. The duration of medication varied from 7 days to 3 months. 20 studies were RCTs, and 2 studies had no Enterococci-related data.

### 3.2 Characteristics of included literature

A total of 20 studies (Yanjie et al., 2008; Hai et al., 2010; Li et al., 2013; Minghong, 2014; Han et al., 2017; Yang et al., 2018; Miao et al., 2019; Wang et al., 2020b; Chen et al., 2020; Gao et al., 2020; Li et al., 2020; Lin et al., 2020; Zhang F. et al., 2020; He et al., 2021; Hu and Hao, 2021; Liu et al., 2021; Song, 2021; Sun et al., 2021; Wang S. et al., 2021; Wu et al., 2021) were included, and 14 RCT experiments of oral medicine use 10 TCM prescriptions: Buzhong yiqi Decoction (BZYQ), Shenling bai zhu Decoction (SLBZ), Danggui buxue Decoction (DGBX), Gegen qinlian Decoction (GGQL), Jishen Decoction (JS), Jianpi jiedu Decoction (JPJD), Jianpi shenshi Decoction (JPSS), Sijunzi Decoction (SJZ), Yiqi jianpi Decoction (YQJP), and Chongjian zhongqi kangai Decoction (CJZQKA). Three RCT experiments used external treatment methods, including Ehuang Decoction (EH) of enema, Xiaozheng Huaji Decoction (XZHJ) of enema, acupoint catgut embedding, skin scraping therapy and ginger separated moxibustion. Three RCT experiments involved TCM injection: Aidi injection. The treatment duration ranged from 7 to 90 days. Table 1 gives the basic information about the involved literature.

**TABLE 1:** Characteristics of the 20 trials included in the network meta-analysis.

Study	Patients		Interventions		Duration (days)	Outcomes							
	IG	CG	IG	CG		Bifidobacterim IG/CG		*Lactobacillus* IG/CG		*Escherichia coli* IG/CG		*Enterococcus* IG/CG	
Sun et al. (2021) China	40	40	BZYQ + CT	CT	90	7.15 ± 0.63	8.77 ± 0.24	7.01 ± 0.45	8.55 ± 0.71	10.76 ± 0.89	9.04 ± 0.63	−	−
Li et al. (2020) China	56	56	SLBZ + CT	CT	84	9.04 ± 0.68	7.10 ± 0.89	9.65 ± 0.64	7.50 ± 0.65	7.56 ± 0.34	9.05 ± 0.41	8.50 ± 0.43	9.93 ± 0.89
Hu and Hao, (2021) China	39	39	DGBX + CT	CT	12	8.5 ± 1.12	7.94 ± 1.05	7.57 ± 1.26	7.00 ± 1.22	8.75 ± 1.35	9.45 ± 1.54	9.03 ± 1.25	9.74 ± 1.34
Yang et al. (2018) China	42	42	Enema therapy with TCM (EHT)+CT	CT	7	8.46 ± 0.71	7.61 ± 0.73	8.25 ± 0.63	7.14 ± 0.55	9.05 ± 0.82	9.73 ± 0.75	7.89 ± 0.67	7.42 ± 0.65
Wang et al. (2020a) China	43	43	Acupoint catgut embedding + CT	CT	21	8.23 ± 0.26	7.38 ± 1.85	8.08 ± 1.96	7.02 ± 1.84	9.04 ± 2.58	10.53 ± 2.96	7.89 ± 1.81	7.14 ± 1.67
Han et al. (2017) China	75	75	GGQLT + CT	CT	84	7.21 ± 0.33	6.56 ± 0.38	6.31 ± 0.22	5.81 ± 0.26	8.25 ± 0.56	9.1 ± 0.47	9.26 ± 0.25	10.21 ± 0.35
He et al. (2021) China	43	43	cutaneous scraping therapy + Moxibustion therapy + CT	CT	21	8.94 ± 0.56	7.91 ± 0.64	8.97 ± 0.46	7.76 ± 0.62	6.23 ± 1.56	8.14 ± 1.32	4.26 ± 0.54	5.93 ± 0.74
Miao et al. (2019) China	30	30	JS + CT	CT	112	9.12 ± 1.11	8.04 ± 0.91	8.29 ± 0.94	7.26 ± 0.76	8.82 ± 0.97	9.39 ± 1.21	8.44 ± 0.91	7.56 ± 0.81
Song et al. (2021) China	75	75	JPJD + CT	CT	21	5.02 ± 1.31	7.62 ± 1.34	5.18 ± 0.13	6.57 ± 0.11	9.58 ± 0.16	8.26 ± 0.17	−	−
Chen et al. (2021) China	28	28	JPJD + CT	CT	56	8.18 ± 1.35	6.54 ± 0.51	7.89 ± 1.41	6.22 ± 0.68	6.21 ± 0.92	8.32 ± 1.41	4.71 ± 0.97	5.95 ± 0.84
Wang J. et al. (2021) China	33	36	JPJD + CT	CT	90	8.30 ± 1.13	7.78 ± 0.97	7.99 ± 0.81	7.60 ± 0.75	6.31 ± 0.97	7.35 ± 0.95	4.81 ± 0.95	5.33 ± 0.97
Hai et al. (2010) China	30	30	JPSS + CT	CT	28	6.86 ± 0.32	5.86 ± 0.32	8.12 ± 0.39	7.33 ± 0.23	7.65 ± 0.18	7.80 ± 0.19	−	−
Zhang F. et al. (2020) China	30	30	SJZ + CT	CT	10	8.12 ± 0.31	5.83 ± 0.36	8.62 ± 0.36	6.94 ± 0.21	7.01 ± 0.15	6.92 ± 0.21	5.94 ± 0.3	5.84 ± 0.25
Wu et al. (2021) China	44	43	SJZ + CT	CT	8	7.59 ± 2.68	5.14 ± 1.48	6.64 ± 2.27	5.38 ± 1.77	6.85 ± 1.66	7.51 ± 2.1	6.30 ± 1.28	7.51 ± 2.1
Lin et al. (2020) China	109	109	Enema therapy with TCM (XZHJ)+CT	CT	7days	8.89 ± 0.74	7.64 ± 0.76	8.28 ± 0.66	7.17 ± 0.58	9.08 ± 0.85	9.76 ± 0.78	7.92 ± 0.7	7.45 ± 0.68
Liu et al. (2021) China	39	39	YQJP + CT	CT	21days	9.96 ± 1.78	8.31 ± 1.12	9.78 ± 1.42	8.32 ± 1.14	4.32 ± 0.56	6.25 ± 0.78	3.21 ± 0.41	4.96 ± 0.65
Gao et al. (2020) China	40	40	CJZQKA + CT	CT	21	7.42 ± 1.28	6.23 ± 1.16	5.27 ± 0.64	4.35 ± 0.67	7.23 ± 1.14	5.07 ± 0.76	6.03 ± 0.72	4.83 ± 0.56
Li (2013) China	30	30	AD injection + CT	CT	-	8.48 ± 0.21	4.84 ± 0.24	7.82 ± 0.34	3.87 ± 0.36	3.05 ± 0.24	5.28 ± 0.29	2.86 ± 0.42	5.98 ± 0.25
Yanjie et al. (2008) China	30	30	AD injection + CT	CT	10	6.73 ± 0.12	6.00 ± 0.36	8.46 ± 0.32	7.49 ± 0.18	7.76 ± 0.21	7.78 ± 0.25	9.07 ± 0.2	8.96 ± 0.22
Minghong (2014) China	30	30	AD injection + CT	CT	10	8.48 ± 0.21	6.94 ± 0.24	7.82 ± 0.34	5.84 ± 0.31	3.15 ± 0.24	5.28 ± 0.29	3.16 ± 0.42	3.25 ± 0.25

Abbreviations: CT, common treatment (radiotherapy-chemotherapy-surgery). Traditional chinese medicine of oral administration: BCYQ, Bu-Zhong-Yi-Qi Decoction; SLBZ, Shen-ling-bai-zhu Decoction; DGBX, Dang-Gui-Bu-Xue Decoction; GGQL, Ge-Gen-Qin-Lian Decoction; JS, Ji-Shen Decoction; PJD, Jian-Pi-Jie-Du Decoction; JPSS, Jian-Pi-Shen-Shi Decoction; SJZ, Si-Jun-Zi Decoction; YQJP, Yi-Qi-Jian-Pi Decoction; CJZQKA, Chong-Jian-Zhong-Qi-Kang-Ai Decoction. External therapy of TCM: EH, Ehuang-Decoction of enema; XZHJ, Xiao-Zheng-Hua-Ji Decoction of enema.

### 3.3 Risk of basis

**FIGURE 2:**
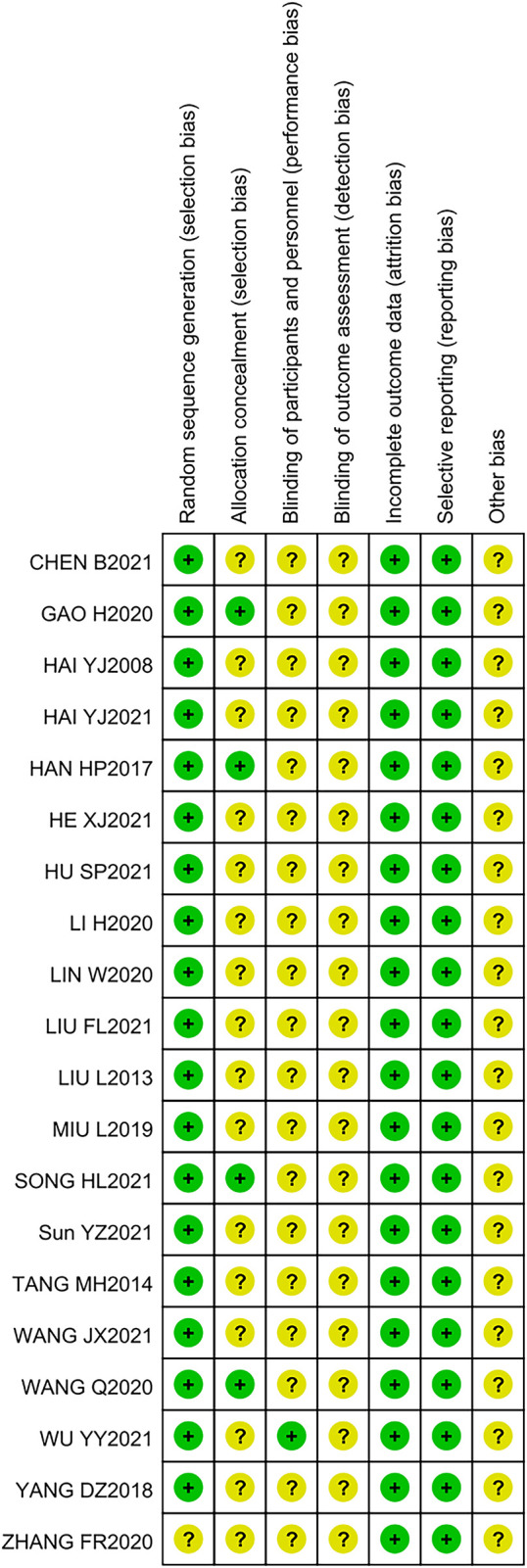
Quality assessment of inclusive literature.

The results of the quality assessment are presented in Figure 2, which shows that the risks of a large proportion of the studies were unclear and low. However, the overall quality of the 20 RCTs was acceptable.

### 3.4 Outcome indicators

#### 3.4.1 Data analysis

**FIGURE 3:**
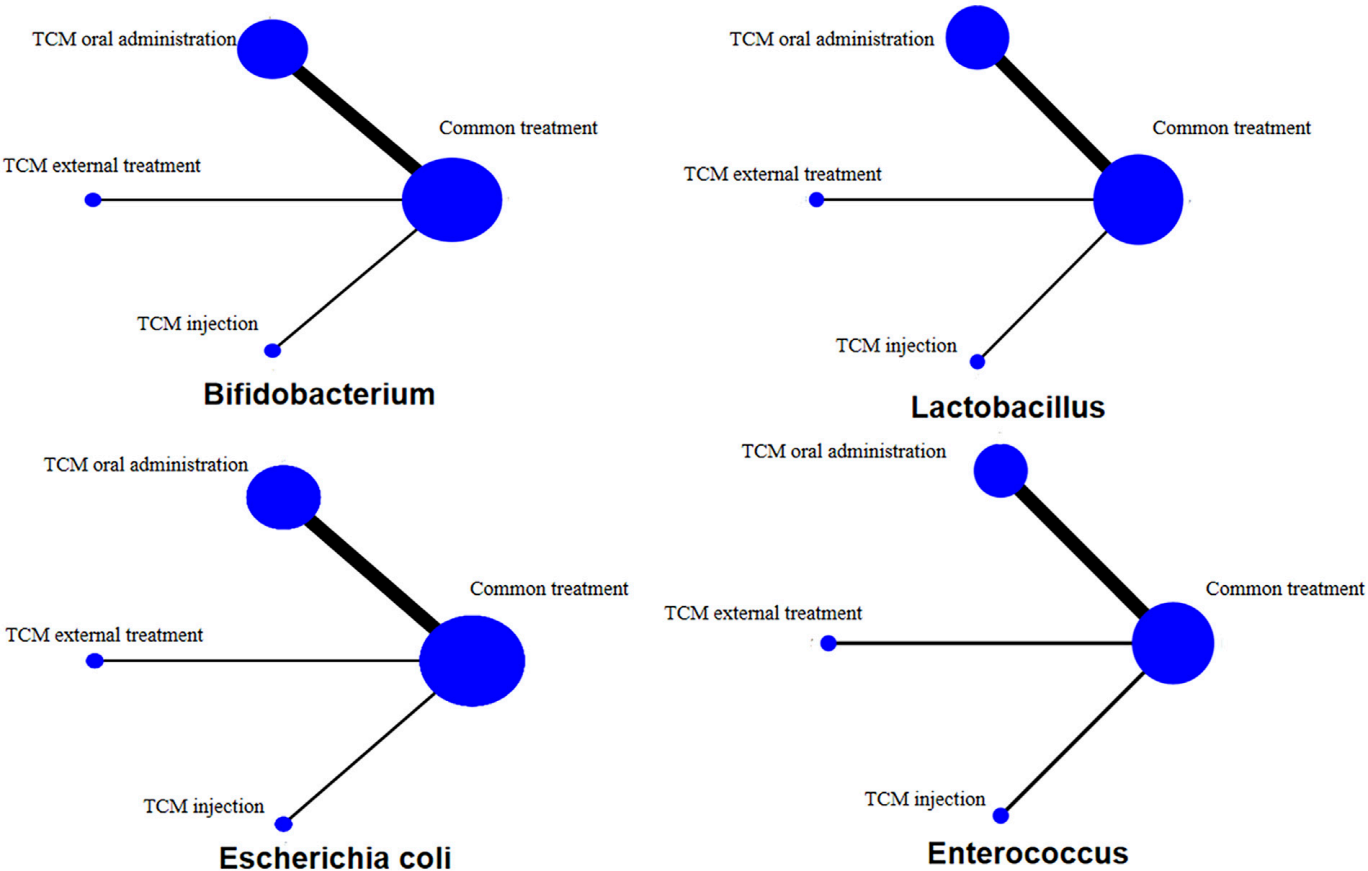
Network diagram.

The network diagram includes 20 RCTs. The line between two points indicates the evidence for direct comparison between the two methods. There is no closed loop between interventions; that is, there is no direct comparison between interventions (Figure 3). Among the four bacteria, the three types of TCM treatment measures (oral administration, external treatment and injection) plus CT are directly compared with CT only, and the thickness of the line indicates the number of RCTs. This shows that the number of treatment methods using oral Chinese medicine is the largest, followed by external treatment and injection. All pairwise comparisons between interventions were from indirect comparisons. Therefore, statistical analysis can be performed directly under the consistency model.

#### 3.4.2 Publication bias

**FIGURE 4:**
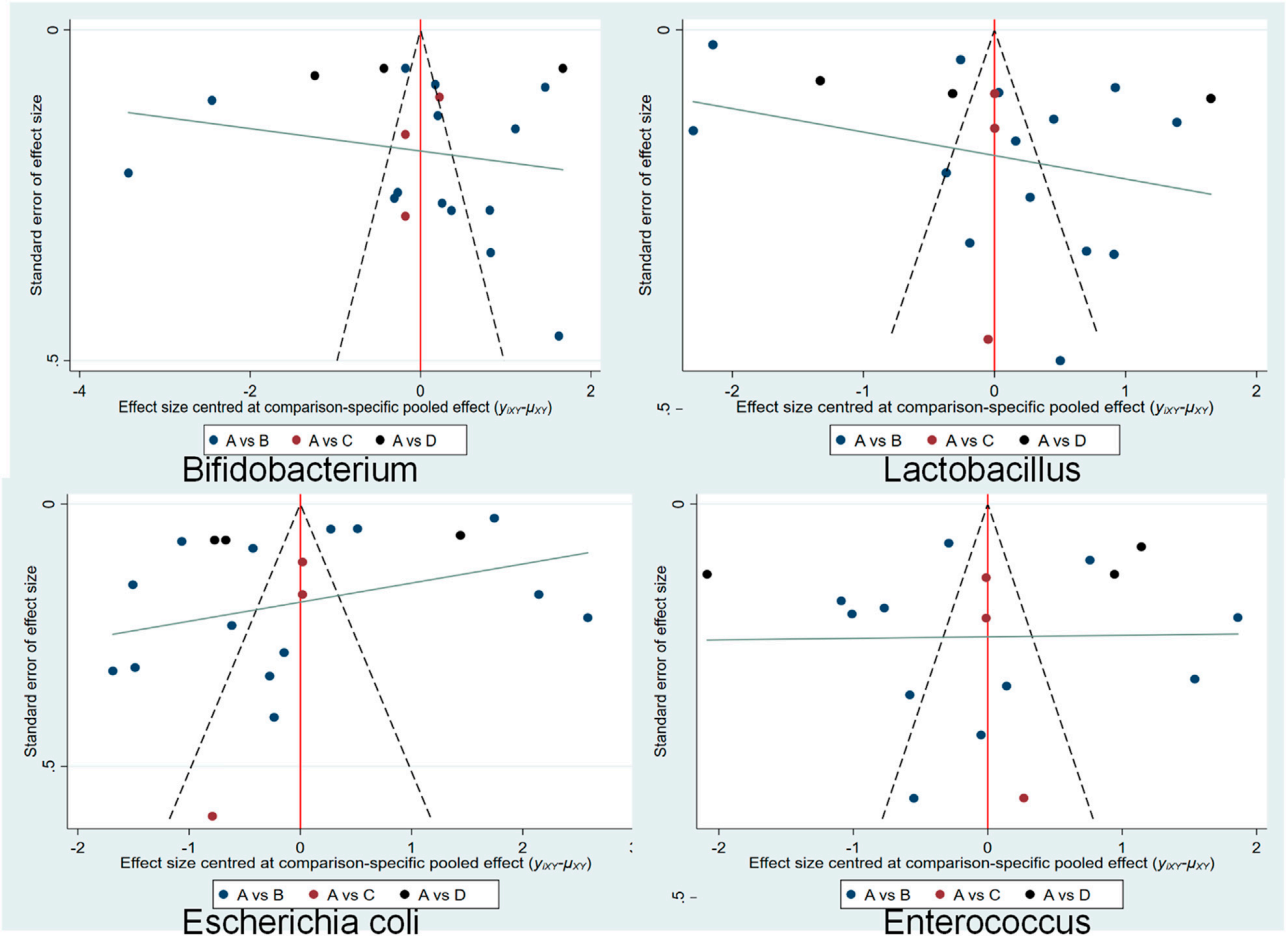
Funnel chart for comparison and correction of bifidobacteria, *Lactobacillus*, *Escherichia coli* and *Enterococcus*. **(A)** conventional treatment, **(B)** conventional treatment + TCM oral administration, **(C)** conventional treatment + TCM external treatment, **(D)** conventional treatment + TCM injection.

Publication bias was assessed by the comparative-adjusted funnel method. Comparative correction charts were prepared for the included studies to evaluate the small sample effects. As shown in Figure 4, the RCTs with *Lactobacillus* and *Bifidobacterium* as outcome indicators in this study are roughly symmetrically distributed on both sides of the midline, indicating that the possibility of a small sample effect is low, and RCTs with *Escherichia coli* and *Enterococcus* as outcome indicators are not symmetrically distributed on both sides of the midline, indicating that the possibility of a small sample effect is high.

#### 3.4.3 Network meta-analysis

In the comparison of pairwise methods, a total of 6 groups are meaningful (Table 2). In the *bifidobacteria* group, there were 2 pairs of comparison with statistically significant differences. CT only was compared with TCM injection in combination with CT, which the MD is 1.97 [MD = 1.97, 95% CI (0.48, 3.46)]. CT only was compared with oral administration of TCM in combination with CT, which MD is 0.83 [MD = 0.83, 95% CI (0.13,1.53)]. In *Lactobacillus*, 3 pairs of comparison showed statistically significant differences. TCM injection plus CT was compared with oral administration of TCM plus CT, which the MD is 1.55 [MD = 1.55 95% CI (0.20, 2.89)]. TCM injection combined with CT only was compared with CT, which the MD is 2.3 [MD = 2.30, 95% CI (1.08, 3.51)]. Oral administration of TCM combined with CT was compared with CT, which the MD is 0.75 [MD = 0.75, 95% CI (0.18, 1.32)]. In *Escherichia coli*, there is a statistically significant difference in one pair of comparison, the curative effect of TCM injection combined with CT was compared with CT only, which the MD is -1.46 [MD = -1.46, 95% CI (- 2.88, - 0.03)]. Among enterococci, one pair of comparison indicated a statistically significant difference. Oral administration combined with CT of TCM Compare with Common treatment, which the MD is -0.66 [MD = -0.66, 95% CI (- 1.31, - 0.01)].

**TABLE 2:** Network meta-analysis matrix of results Comparison of treatments: Mean difference (95% confidence intervals).

Bifidobacterium			
TCM injection treatment + CT			
0.98 (−1.13, 3.10)	TCM external treatment + CT		
1.14 (−0.51, 2.79)	0.16 (−1.50, 1.82)	TCM oral prescription + CT	
1.97 (0.48, 3.46)	0.99 (−0.52, 2.49)	0.83 (0.13, 1.53)	Common treatment
*Lactobacillus*			
TCM injection treatment + CT			
1.20 (−0.53, 2.94)	TCM external treatment + CT		
1.55 (0.20, 2.89)	0.34 (−1.03, 1.71)	TCM oral prescription + CT	
2.30 (1.08, 3.51)	1.09 (−0.15, 2.34)	0.75 (0.18, 1.32)	Common treatment
*Escherichia coli*			
TCM injection treatment + CT			
−0.54 (−2.59, 1.51)	TCM external treatment + CT		
−1.03 (−2.61, 0.54)	-0.49 (−2.11, 1.13)	TCM oral prescription + CT	
−1.46 (−2.88, −0.03)	-0.92 (−2.39, 0.56)	−0.43 (−1.09,0.24)	Common treatment
*Enterococcus*			
TCM injection treatment + CT			
−1.59 (−3.35, 0.17)	TCM external treatment + CT		
−0.37 (−1.76, 1.02)	1.22 (−0.20, 2.63)	TCM oral prescription + CT	
−1.03 (−2.26, 0.20)	0.56 (−0.70, 1.81)	0.66 (−1.31, −0.01)	Common treatment

Abbreviations: TCM, traditional chinese medicine; CT, common treatment.

#### 3.4.4 SUCRA probability ranking

**FIGURE 5:**
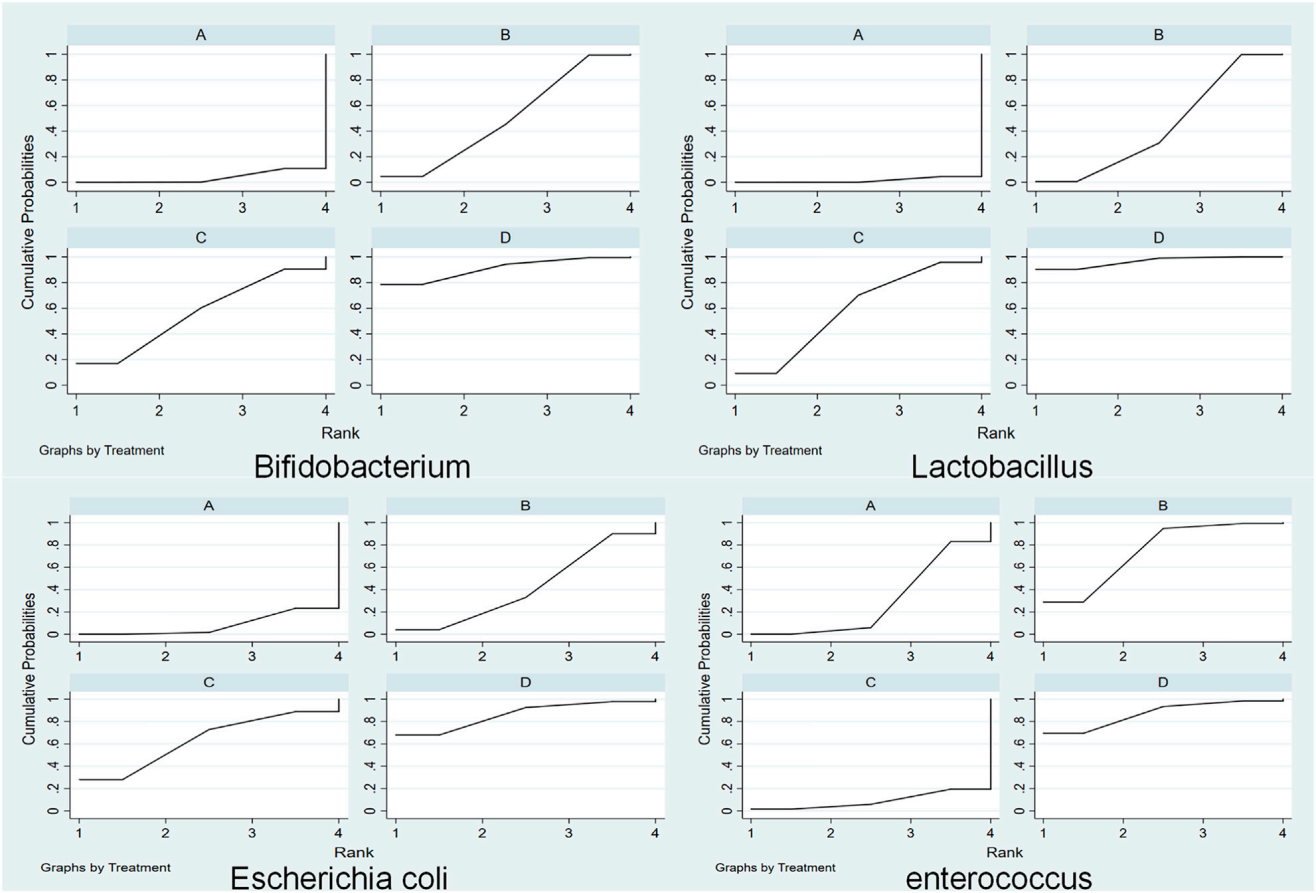
Cumulative probability of total effective rate (Abbreviations: **(A)** traditional therapy; **(B)** traditional therapy + traditional Chinese medicine (TCM) oral prescription; **(C)** common treatment + TCM external treatment; **(D)** common treatment + TCM injection treatment).

The cumulative ranking of the four methods is shown in Figure 5. High SUCRA values are correlated with good efficacy of this treatment for this type of gut microbiota. According to Sucra values, the total ranking of the four methods (A, Common Treatment; B, Common treatment plus TCM oral prescription; C, Common treatment plusTCM external treatment; D, Common treatment + TCM injection treatment) in supporting *bifidobacteria* was: D (Sucra = 90.08) > C (Sucra = 55.9) > B (Sucra = 49.7) > A (Sucra = 3.6); in supporting *Lactobacillus*, the total ranking of four methods was: D (Sucra = 96.4) > C (Sucra = 58.4) > B (Sucra = 43.7) > A (Sucra = 1.5); for *Escherichia coli*, the total ranking was: D (Sucra = 86.1) > C (Sucra = 63.2) > B (Sucra = 42.4) >A (Sucra = 8.3); and in inhibiting *Enterococcus*, the total ranking was: D (Sucra = 87.1) > B (Sucra = 74.3) > A (Sucra = 29.6) > C (Sucra = 8.9); To sum up, CT plus TCM injection can increase the probiotics (bifidobacteria and lactic acid bacteria) and reduce the pathogens (*Escherichia coli* and *Enterococcus*) in the intestinal tract of patients with gastric cancer. Thus, it is the best choice.

#### 3.4.5 Comparative effect of colorectal cancer and gastric cancer

To make the results more stable and credible, we performed a meta-analysis supplementing the intestinal flora of colorectal and gastric cancers (Table 3). The random-effects model shows that TCM injection plus CT (WMD = 1.97, 95% CI (0.273, 3.667), *p* < 0.05] or TCM external treatment plus CT (WMD = 1.046, 95% CI (0.843,1.249), *p* < 0.05] is compared with CT, the amount of *Bifidobacteria* and *Lactobacillus* in feces of colorectal cancer patients are higher than those of the control group. For *Escherichia coli*, the amount of *Escherichia coli* in colorectal cancer patients treated with external treatment plus CT (WMD = -1.070, 95% CI (- 1.579, - 0.561), *p* < 0.05] is lower than that in the control group. As compared to CT only, colorectal cancer patients treated with TCM external therapy plus CT (WMD = 0.627,95% CI (0.35,0.904), *p* < 0.05] have significant differences in *Enterococcus* in the treatment group. Conclusion: compared with CT only, the combination of TCM injection or external treatment with CT is more effective for supporting the number of *Bifidobacterium* and *Lactobacillus* in colorectal cancer. Similarly, as compared to CT, the effect of external treatment of TCM combined with CT is better for inhibiting the number of *Enterococcus* and *Escherichia coli* in intestinal cancer.

**TABLE 3:** Comparative Effect of Colorectal Cancer and Gastric Cancer in the meta-analysis.

Outcome	Disease	Treatment	WMD	[95% Conf. interval]		p
Bifidobacterium	Colorectal cancer	TCM oral treatment + CT vs. CT	0.648	−0.114	1.409	0.096
Bifidobacterium	Colorectal cancer	TCM injection + CT vs. CT	1.970	0.273	3.667	0.023
Bifidobacterium	Colorectal cancer	TCM external treatment + CT vs. CT	1.046	0.843	1.249	0
Bifidobacterium	Gastric cancer	TCM oral treatment + CT vs. CT	0.975	−1.269	3.218	0.394
*Lactobacillus*	Gastric cancer	TCM oral treatment + CT vs. CT	0.650	−0.384	1.683	0.218
*Lactobacillus*	Colorectal cancerr	TCM injection + CT vs. CT	2.299	0.605	3.994	0.008
*Lactobacillus*	Colorectal cancer	TCM external treatment + CT vs. CT	1.135	1.017	1.252	0
*Lactobacillus*	Colorectal cancer	TCM oral treatment + CT vs. CT	0.691	−1.174	2.557	0.468
*Escherichia coli*	Colorectal cancer	TCM oral treatment + CT vs. CT	−0.475	−1.182	0.232	0.188
*Escherichia coli*	Colorectal cancer	TCM injection + con CT CT	−1.460	−2.934	−0.015	0.052
*Escherichia coli*	Colorectal cancer	TCM external treatment + CT vs. CT	−1.070	−1.579	−0.561	0
*Escherichia coli*	Gastric cancer	TCM oral treatment + CT vs. CT	0.436	−1.734	2.606	0.694
*Enterococcus*	Colorectal cancer	TCM oral treatment + CT vs. CT	−0.402	−1.005	0.201	0.191
*Enterococcus*	Gastric cancer	TCM oral treatment + CT vs. CT	−0.471	−2.440	1.499	0.693
*Enterococcus*	Colorectal cancer	TCM external treatment + CT vs. CT	0.627	0.350	0.904	0
*Enterococcus*	Gastric cancer	TCM injection + CT vs. CT	−1.033	−2.969	0.903	0.296

Abbreviations: CT, common treatment; adiotherapy-chemotherapy-surgery. TCMoraltreatment; BZYQ, Bu-Zhong-Yi-QiDecoction; SLBZ, Shen-ling-bai-zhu Decoction; DGBX, Dang-Gui-Bu-Xue Decoction; GGQL, Ge-gen-Qin-lian Decoction; JS, Ji-Shen Decoction; JPJD, Jian-Pi-Jie-Du Decoction; JPSS, Jian-Pi-Shen-Shi Decoction; SJZ, Si-Jun-Zi Decoction; YQJP, Yi-Qi-Jian-Pi Decoction; CJZQKA, Chong-Jian-Zhong-Qi-Kan-Ai Decoction. TCM, injection, Eddie injection; TCM, external treatment; EH, Ehuang Decoction of enema; XZHJ, Xiaozheng Huaji Decoction of enema acupoint catgut embedding, cutaneous scraping therapy and ginger separated moxibustion.

## 4 Discussion

A total of 20 RCTs with 1774 patients was included in this paper. Through the comparison of SUCRA results, Aidi injection is the most effective in increasing the number of *Bifidobacteria* and *Lactobacillus*, and in inhibiting the number of *Escherichia coli* and *Enterococcus*. In the pairwise comparison of three TCM treatments, injection of TCM plus CT or oral Chinese medicine plus CT are effective for *Bifidobacteria* and *Lactobacillus*. For *Escherichia coli*, TCM injection plus CT takes effect. For *Enterococcus*, TCM oral treatment plus CT is practical.

There are three treatments of TCM, including internal treatment (oral absorption), external treatment (physical therapy or skin mucosal absorption), and injection (direct blood injection). Internal treatment mainly uses oral Chinese medicine decoction, the preparation of which is to soak the traditional Chinese medicine in boiling water or hot water to produce an aqueous extract containing a mixture of chemical components (Zhou et al., 2016; Deng et al., 2019). Specifically, after oral administration of TCM into the colon, intestinal microbiota converts carbohydrates, proteins, lipids and small non-nutritive compounds from TCM into chemical metabolites that may have beneficial or adverse effects on human health (Wang et al., 2013). For example, the continuous digestion of polysaccharides and carbohydrates (PS) produces many short-chain oligosaccharides, which can promote the growth of probiotics such as *Bifidobacteria* and *Bacteroides*. Shorter PSs are digested to form monosaccharides, which can be continuously catabolized to form short-chain fatty acids (SCFA) (e.g., formate, acetate, propionate, butyrate), lactic acid, hydrogen, carbon dioxide and other metabolites. Valproic acid, a kind of SCFAs, has antitumor activity, and its main mechanism is to inhibit histone deacetylase (Gurvich et al., 2004). These metabolites may directly affect the host intestinal environment and improve the microenvironment of gastrointestinal cancer (Vernocchi et al., 2016; Feng et al., 2019).

Aidi injection is mainly composed of ginseng, *Astragalus* membranaceus, canthatis, and acanthopanax senticosus. The active components are ginsenoside, astragalus polysaccharide, astragalus saponin, cantharidin and Acanthopanax Senticosus Polysaccharide (Quirke et al., 2007). *Astragalus* polysaccharides can increase the number of *lactic acid bacteria* and *Bifidobacteria*, thereby reducing pro-inflammatory factors, such as interleukin-6 and tumor necrosis factor-α. Therefore, as an inflammatory response inhibitor, it can also reduce the inflammatory response by reducing *Salmonella typhi* in the intestine (Tang et al., 2021). Ginsenoside-rb3 and ginsenoside Rd can promote the growth of beneficial bacteria, such as *Bifidobacterium*, *Lactobacillus*, *Acidophilus* and *Anisoid*, and can also reduce a number of cancer-related pathogens and *Helicobacter pylori spp* to prevent the development of colorectal cancer (CRC) (Huang et al., 2017). There are few studies on other drugs. We attribute the better effect of Aidi injection to its high bioavailability compared with the other two methods. When it comes to the cold and hot nature of the drug, all four drugs in the prescription are warm products. Therefore, it can be inferred that Aidi injection is hot and may be more suitable for the body of cancer patients undergoing radiotherapy and chemotherapy. External treatments include enema, acupoint embedding, skin scratching and ginger moxibustion. These methods are rarely studied in the field of intestinal flora research.

Many studies have shown that there is a causal relationship between changes in the intestinal flora and colorectal cancer. Patients with colorectal cancer have poor nutritional status and low systemic and partial resistance, which inhibit the growth of intestinal dominant bacteria, such as *Lactobacillus* and *Bifidobacterium*, resulting in the imbalance of the intestinal microenvironment. Meanwhile, intestinal flora imbalance will decrease the immune function of the body, and the decline of immune function will aggravate the flora imbalance, thus forming a vicious circle. In addition, chemotherapy drugs further reduce the immunity of patients and interfere with the proportion of normal intestinal flora. Moreover, the more obvious the imbalance is before chemotherapy, the more serious the imbalance is after chemotherapy. Therefore, the anti-cancer research of intestinal flora is of great significance. *Lactic acid bacteria* and *Bifidobacterium* strains induce dendritic cell (DC) to mature (Hickey et al., 2021) and produce IFN- γ (IFN- γ), enhancing the cytolytic potential of NK cells (Zhou et al., 2019). Probiotics induce apoptosis by inhibiting the expression of COX-2, NF KB, and MAPK, suppressing the inactivation of inflammatory bodies, and activating Caspase-3 (Iyer et al., 2008). It also induces cell death through autophagy (Engevik et al., 2019). *Bifidobacterium* can increase anti-PD-L1 and inhibit tumor volume by inducing anti-inflammatory activity of macrophages and dendritic cells (Xu et al., 2020). Probiotics such as *Lactobacillus* and *Bifidobacterium* inhibit the growth of colorectal cancer by suppressing inflammation and angiogenesis, and enhance the intestinal barrier function by secreting short-chain fatty acids (SCFAs) (Koh et al., 2016). *Escherichia coli* is more prevalent in colorectal cancer tissues (Buc et al., 2013). *Enterococcus faecalis* produces enterotoxins (e.g., tartary buckwheat glucoside) and reactive oxygen species, which can lead to DNA oxidative damage and intestinal epithelial cell inflammation (Baldassarri et al., 2005). *Enterococcus faecalis* is responsible for producing reactive oxygen species and superoxide anions, resulting in DNA damage and genomic instability in colorectal cancer (Geravand et al., 2019). Fecal *Escherichia coli* induces mucosal macrophages to produce DNA damage inducers (Goodwin et al., 2011), such as 4-hydroxy-2-nonyl, through COX-2 (Yang et al., 2013).

We did a general meta-analysis, separating the patients with gastric cancer and colorectal cancer, to compare the differences between CT only and the three methods combined with CT. For colorectal cancer patients, in *Bifidobacteria* and *Lactobacillus*, external treatment plus CT or injection of TCM plus CT, are more effective than CT. For *Escherichia coli*, the TCM external treatment plus CT and the TCM injection plus CT are more effective than CT only. For *Enterococci*, We prefer TCM injection plus CT because it exhibits better effect than CT only.

## 5 Limitations

The following limitations should be considered in this study. The methodological quality of the effect of TCM on intestinal flora of gastrointestinal cancers is subject to some risk deviation, such as insufficient sample size and short duration. In addition, gastrointestinal cancers are subdivided into gastric and colorectal cancers. Because the number of articles is too small to perform a heterogeneity testing, we did a general meta-analysis to assist the results. TCM is divided into three categories. The external treatment includes enema, the effect of which overlaps with that of oral TCM. However, considering its direct effect on intestinal flora and its short duration, it is placed in the external treatment.

## 6 Conclusion

In this study, *Bifidobacteria* and *Lactobacilli*, *Escherichia coli* and *Enterococci* were used as the main therapeutic indicators for comprehensive evaluation. Overall, TCM injection may be the best treatment, followed by TCM external treatment. TCM plays a certain role in the intestinal flora of patients with gastrointestinal cancers through multi-targeted comprehensive intervention. Clinically, it can be used in combination with other therapies depending on the actual situation of patients, and is suitable for the whole treatment process for gastrointestinal cancer patients.

## Data Availability

The original contributions presented in the study are included in the article/supplementary material, further inquiries can be directed to the corresponding authors.
